# Mathematical Modeling of *Escherichia coli* and *Lactobacillus acidophilus* Growth Based on Experimental Mixed Batch Cultivation

**DOI:** 10.3390/ijms262311493

**Published:** 2025-11-27

**Authors:** Gabriela Isopencu, Valentina Gogulancea, Vasile Lavric, Ionut Banu

**Affiliations:** 1 Department of Chemical and Biochemical Engineering, National University of Science and Technology POLITEHNICA Bucharest, 1-7 Gh. Polizu, 011061 Bucharest, Romania; gabriela.isopencu@upb.ro (G.I.); vasile.lavric@upb.ro (V.L.); 2 School of Computing, Engineering and Intelligent Systems, Ulster University, Derry~Londonderry Campus, Londonderry BT48 7JL, UK; v.gogulancea@ulster.ac.uk

**Keywords:** bacterial mixed culture, cell viability, flow cytometry, time scale, segregated mathematical model

## Abstract

A better understanding of cultivation of microorganisms in mixed culture is needed to identify the relationships between different bacterial strains. *Lactobacillus acidophilus* (ATCC 4796) Gram-positive bacteria and *Escherichia coli* (K12-MG1655) Gram-negative bacteria are two microorganisms that can interact accidentally in the dairy food chain process or in different human pathologies. This work focused on how bacterial populations evolve in batch culture, depending on the nature of the carbon source, by monitoring cell viability using flow cytometry and substrate concentration. The experiments monitored the time evolution of bacterial populations grown on two different cultivation media (single source of carbon—SSCM and de Man, Rogosa and Sharpe—MRS broth media) which stimulated different proliferation conditions. Experimental data were used to calibrate a segregated mathematical model (accounting for two bacterial strains—biological clusters—with their individual birth time, an event that creates a new timeline cluster to which daughters belong) that highlights in silico the various interactions that can occur between two bacterial species.

## 1. Introduction

Cultivation techniques are particularly important for in vitro studies of multi-specific cell consortia that reproduce natural systems [1,2,3]. The potential for designed cell-based systems has increased due to advancements in metabolic engineering and synthetic biology [4,5]. The engineering of consortia, in which populations of different cells cooperate to carry out a specific function, is currently an emerging field of interest, and cell population control is necessary for this approach [6,7]. The interactions identified in experimental research involving multi-cellular consortia are integrated into fundamental research to develop new directions in industrial, biotechnological, and environmental technologies [8,9]. Co-culture model systems, such as tissue or ecological niches, are highly complex and have random interactions at different timescales and space scales. Therefore, many synthetic biological co-culture systems are developed for future industrial, agricultural, or environmental applications [10,11], such as cell–cell communication [12], switching for population control [13,14], designing bacterial cells for killing pathogens [15,16], food utilization [17], cellular growth control in plant fermentations [18,19,20], biosensor design [21], microbial fuel cells [22], and production of chemicals [23,24,25].

Experimental studies are needed to monitor complex cellular consortia and understand their interactions. They must provide large-scale data acquisition [26], as well as rigorous control over the temporal variation in experimental parameters [15].

Studies in the literature have shown that assessment of the physiological stages of individual bacterial cells (cultivated in laboratory scale environments) can be useful to estimate the behavior of bacterial populations on an industrial scale [12,27].

Bacterial cells at different stages of the cell cycle respond individually to the action of external stimuli in the environment, which results in obtaining heterogeneous/segregated populations in terms of cell age [13,28]. Each bacterial cell has its own metabolic system and responds accordingly when stochastic events take place. So, even in the case of an isogenic population of bacteria growing in apparently homogeneous conditions, small values for nutrient amounts, pH, or oxygen gradients can induce specific changes at a single-cell level. Also, experimental studies have shown that after a disturbance applied to a bacterial culture by an external stimulus, only a fraction of the population responds to this immediate change (those in the appropriate phase of the cell cycle), with the rest providing a secondary response (as is likely the case, in the latency phase) after the occurrence of the disturbance [29,30]. Depending on the specific growth variables of the bacterial species, the correct mechanism is the inherent response of bacterial cells, which can be slow growth or rapid adaptation to induced perturbations [31,32].

There are numerous studies that highlight the probiotic effect of lactic acid bacteria on pathogenic microorganisms [33,34,35] and biofilm formation in medical [8,36,37] and food chains [13,38,39]. Among lactic acid bacteria, *Lactobacillus acidophilus* is frequently analyzed in inhibition studies of various pathogens such as *Escherichia coli* [37,40], *Listeria monocytogenes* [37], *Pseudomonas aeruginosa* [40], and *Staphylococcus aureus* [40] due to the production of lactic acid, but also of secondary metabolites such as surlactin [41] and acidophilin [42] as biosurfactants in native environment.

The concept of time segregation, applied in the present research, is based on a previous work [43] and considers a cell’s birth as a discrete event, assuming that when the cells reach a certain mass during growth, they divide in half. After division, the mother cell continues its timeline, while the daughter starts its own timeline. The process of aging (accumulation of damaged genetic material in mother cells, implying a continuous decline of metabolic activity) is disregarded in the current research, but it can be easily added. Therefore, mother and daughter will then grow and reach another division step after the same period, when they give birth to two identical daughters (no aging), and these progenies form a new cluster. Each new division will generate a new cluster of last birth cells, which starts a new timeline, while the old clusters continue theirs. This way, a temporal segregation appears, together with the synchronicity of the population [43].

This experimental study focused on collecting experimental data for a mixed culture of two bacterial strains, *Escherichia coli* and *Lactobacillus acidophilus*. The research aimed to identify the type of interaction between the two strains, under different conditions, after a preliminary study of the development dynamics of each strain under the same cultivation conditions. The following parameters were measured: cell viability and cell differentiation, which were evaluated by a flow cytometry technique [44,45]. A complex mathematical model, based upon the timeline segregation concept, is built, accounting for two bacterial strains—biological clusters—with their specific birth time, an event that creates a timeline cluster to which daughters belong. The mother (generation 1, inoculum)–daughter (generation 2) synchronicity means that both will give birth, after the same growth time, to daughters (generation 3), with identic growth time. Thus, the fourth generation of daughters will appear after simultaneous birth of generation 1 to 3. This mathematical model could highlight the in silico interactions that can occur between two bacterial species, both segregated in age clusters, according to their timelines, an approach which has a high degree of novelty.

The experimental results were used to determine, through a regression analysis, the parameters of this new segregated mathematical model for the growth of this mixed population. To simplify both the mathematical model of mixed culture growth with direct interest in the probiotic bacteria and the experimental conditions, the simplest carbon sources for both microorganisms—glucose and lactose, respectively—were chosen.

## 2. Results

The microorganisms (*E. coli* and *L. acidophilus*) were grown individually in batch culture to a constant optical density, thus determining the maximum cell concentration possible to obtain on the specific culture medium. The correlation between optical density and cell concentration was assessed based on the standard growth curve of the microorganisms on different cultivation media and flow cytometric analysis.

**Figure 1:**
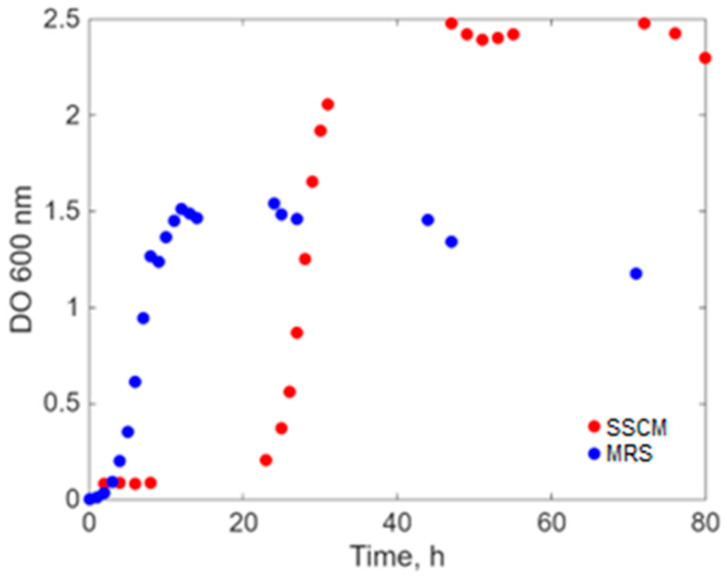
Turbidity of *E. coli* broth cultivated on two different media.

The experimental determinations regarding the cell growth curve of *E. coli* on the two different cultivation media, in batch process, are presented in Figure 1.

**Figure 2:**
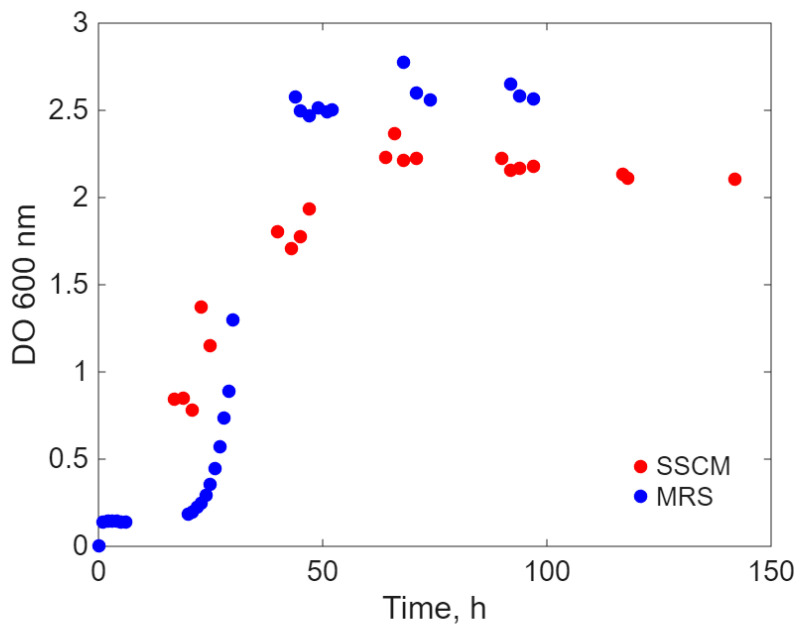
Turbidity of *L. acidophilus* broth cultivated on two different media.

From the growth curve presented in Figure 1, it is observed that *E. coli* has a longer latent phase on MRS (more than 20 h), indicating the fact that the microorganism does not have the appropriate enzymatic systems for this culture medium, with the latter being non-specific to it (Figure S2a,b for total and live/dead cells). After the latent phase, *E. coli* has an almost asymptotic response to the ordinate exponential growth and reaches the plateau phase at an OD 600 density of 2.5, although the actual concentration of live cells in the culture medium does not exceed 1 g/L (Figure S3b). On the synthetic medium—general SSCM—the concentration of viable *E. coli* cells reaches the approximate value of 3.5 g/L (Figure S3a). On this medium, a shorter lag phase is observed (of only 5 h), and the stationary phase is reached at an OD 600 density of about 1.5, being much longer than on the MRS cultivation medium, with a better cell viability yield. For lactic acid bacteria, the growth curve on the two different cultivation media, in batch conditions, is represented in Figure 2.

Figure 2 shows that *L. acidophilus* has a long latent period (also about 20 h) on both SSCM and MRS cultivation media. Also, the cytograms corresponding to live/dead cell growth and differentiation (Figure S4a,b) are influenced by the variation in lactobacilli size in different growth stages (Figure S4b). The differences between the two growth curves, corresponding to the two different cultivation media, are as follows: for MRS, the growth rate in the exponential phase is very high, almost asymptotic to the ordinate, and the stationary/plateau phase is shorter, with a concentration of viable cells of 1.3 g/L (Figure S5b—blue dots), for an OD 600 optical density about 2.75, while on SSCM medium, the exponential phase occurs at a slower rate, allowing for a longer stationary phase, leading to a concentration of viable cells of 2.5 g/L (Figure S5a blue dots), for an optical density of OD 600 about 2.5.

From the results obtained from the individual cultivation of the analyzed microorganisms, the co-culture strategy of their cultivation on the two different cultivation media was identified.

a. Simultaneous inoculation and cultivation on the two different culture media (***Sync-co***), with the same inoculum concentration.
b. Inoculation with *E. coli* after the lag period (approximately 20 h) of *L. acidophilus* only on the SSCM culture medium (***Lag-Sync***) to test the probiotic’s capacity to cope with the proliferation of the pathogenic microorganism on a culture medium favorable to *E. coli*.

The staggered inoculation aimed to test the survival of the pathogen when inoculated with lactic acid bacteria immediately after the end of its latent phase.

**Figure 3:**
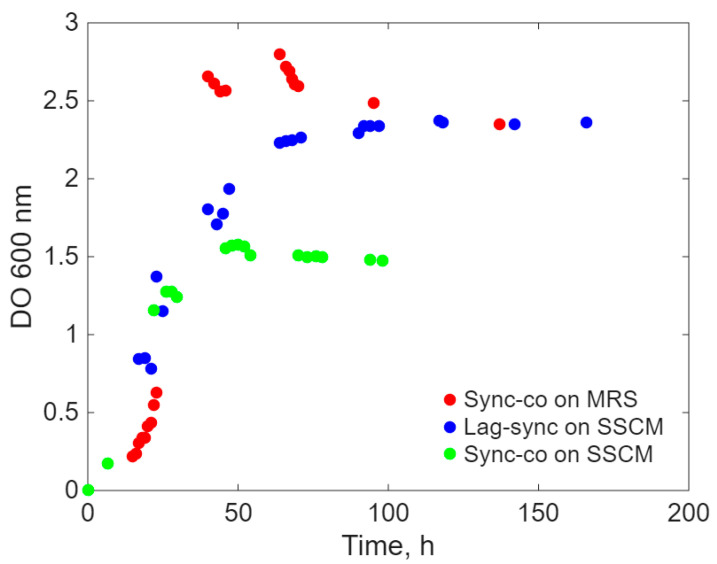
Growth curves of the mixed culture.

The growth curves for the mixed population of the two microorganisms studied are shown in Figure 3. The experimental points obtained for the growth curve upon simultaneous inoculation on SSCM (green dots) show a short lag period, exponential growth with a lower rate, and an intermediate stationary phase with the lowest accumulation of bacterial biomass in the bioreactor (approx. 0.88 g/L—Figure S6a). On the other hand, the experimental points obtained for the growth curve upon simultaneous inoculation on MRS (red dots) show a shorter lag period (approximately 15 h), the same exponential growth almost asymptotic to the ordinate, and a shorter stationary phase with a high accumulation of bacterial biomass in the bioreactor (approx. 7.5 g/L—Figure S6b).

The growth curve of the mixed population on SSCM, obtained by staggered inoculation of the two microbial species (Figure 3—blue dots), shows intermediate values for the lag and exponential phases, as well as for the total concentration of accumulated bacterial biomass (approx. 4.5 g/L—Figure S6c). However, this type of cultivation has the longest stationary phase of the growth curve.

**Figure 4:**
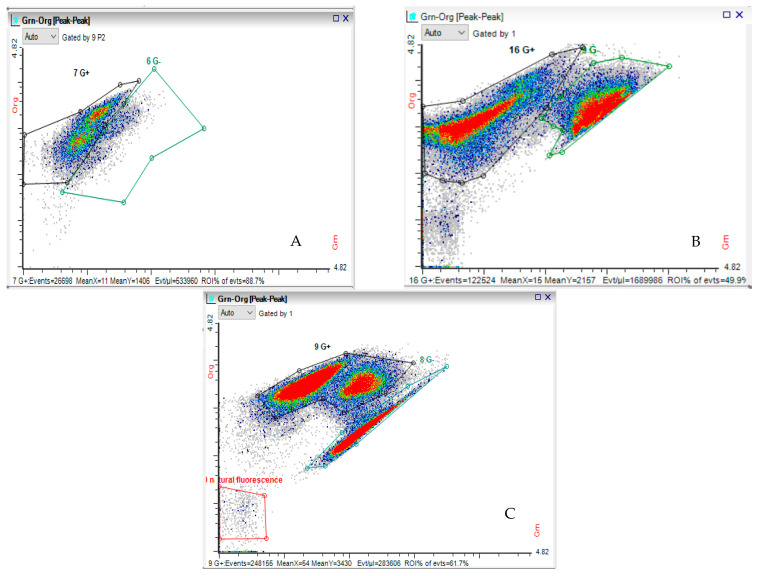
The cytograms for the three co-cultivation ways at final stage of experiment stained with Hexidium Iodide (**A**) **Sync-co** on MRS; (**B**) **Lag-sync** on SSCM; (**C**) **Sync-co** on SSCM. (Full text in the top rectangle: “natural fluorescence”).

*Cell differentiation.* The cytograms used to identify the concentration of individual cells from the two different strains, in mixed culture, are presented in Figure 4. The cytograms presented in Figure 4 aim to highlight the two categories of bacteria (Gram-positive, delimited by black lines, and Gram-negative, delimited by green lines, respectively). Areas with a high cell concentration are marked by an intense red color. The appearance of two concentrated areas of Gram-positive bacteria in Figure 4C indicates two populations of different sizes, assimilated with two different generations.

### Modeling Results

The proposed mathematical model was regressed over the experimental data for all case studies to find the model parameters which minimized the objective function, namely the mean root square of the residual model experiment for the living cell concentration (Figure S1).

The kinetic parameters determined from the regression analysis (for individual cultures) are presented in Table 1, together with the values of the associated objective functions. The modeling results support the experimental findings, showing higher values for the affinity constants in the case of SSCM cultivation for both populations, which indicates a slower growth on the SSCM medium. In addition to this, the experimental data show that the *L. acidophilus* strain grows faster and to higher concentrations in individual culture compared to the *E. coli* strain. This is reflected in the values of both the affinity constants and the maximum specific growth rates.

The standard deviations associated with genetic algorithms are not readily available; therefore, the following strategy was chosen to compute them. Ten runs of the regression analysis were performed, five starting from the same initial values for the parameters, but seeding, randomly, the random number generator, while the other five started from different initial values, but the random number generator started from the same seed. The results are used to compute the average and the standard deviation for each parameter (see Table 1).

The values of the maximum specific growth rates, which are higher for *L. acidophilus* in individual culture, could induce the idea that *E. coli* would be outcompeted by *L. acidophilus* in a mixed culture, considering C-source limitation. Also, the higher substrate affinity constant value for *L. acidophilus* in individual culture on SSCM cultivation media should be highlighted, which indicates the compatibility of this carbon source with lactic bacteria. The values of the inhibition constant, determined via regression analysis using the experiments performed with these two strains on the MRS media (which appears to be unfavorable to both strains), were then used to simulate the behavior of the two strains in synced cultivation on the same cultivation media.

**Figure 5:**
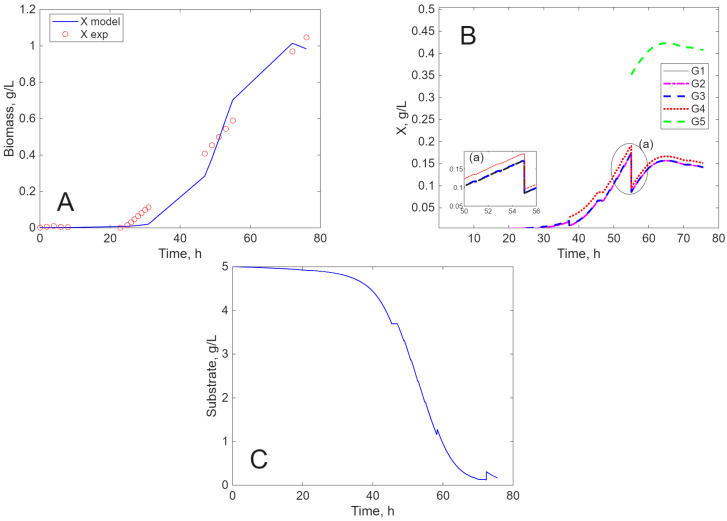
Experimental and simulated results for *E. coli* on SSCM ((**A**)—biomass concentration, (**B**)—cell generations, and (**C**)—substrate concentration).

**Figure 6:**
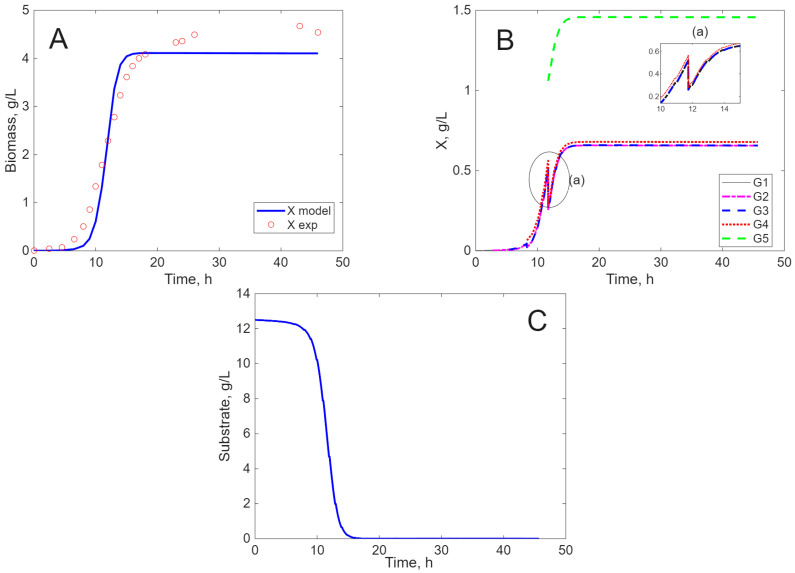
Experimental and simulated results for *E. coli* on MRS ((**A**)—biomass concentration, (**B**)—cell generations, and (**C**)—substrate concentration).

**Figure 7:**
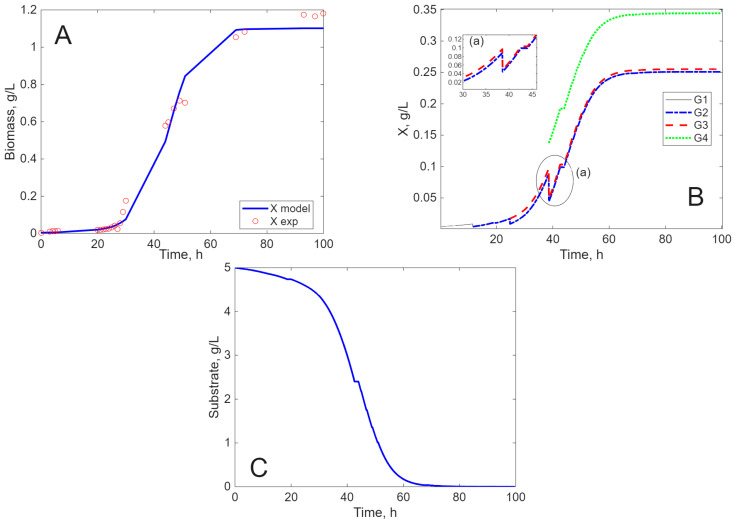
Experimental and simulated results for *L. acidophilus* on SSCM ((**A**)—biomass concentration, (**B**)—cell generations, and (**C**)—substrate concentration).

**Figure 8:**
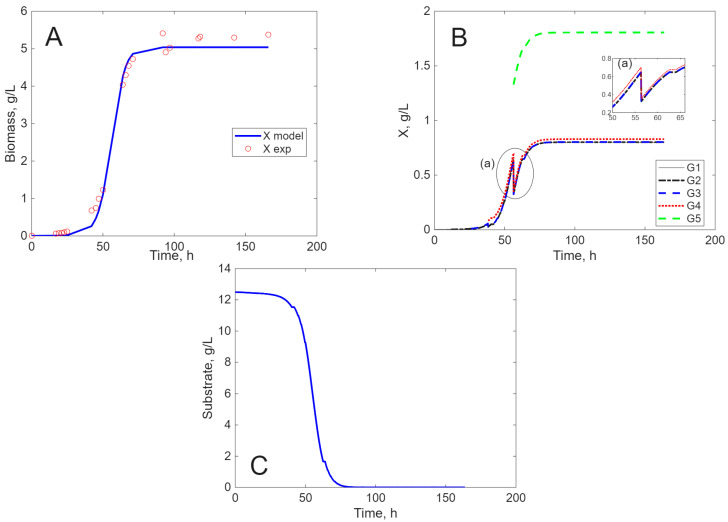
Experimental and simulated results for *L. acidophilus* on MRS ((**A**)—biomass concentration, (**B**)—cell generations, and (**C**)—substrate concentration).

The results of the simulation are presented in Figure 5, Figure 6, Figure 7 and Figure 8, in comparison to experimental data, for biomass concentration, cell generations (as clusters), and substrate consumption (see Section 4.4 Mathematical Modeling—the hypotheses on which it was built, for a better understanding of these figures). Label A shows the calculated (line) and measured values of the concentration of viable biomass produced over time. In Label B, the calculated profiles of cell generations are presented, starting with inoculum (G1) and ending with the last generation (G3, G4, or G5) depending on the cultivation envisaged. Label C shows the mathematical model’s estimate of substrate consumption.

*E. coli* cultivated on SSCM present a large lag period (approx. 20 h) (Figure 5A), followed by the growth of five generations (Figure 5B). Since the aging is disregarded, the generations are synchronized, so they divide at the same time—therefore, all three generation will contribute to the birth of the fourth generation, and then all four generations will contribute to the birth of the fifth generation. Therefore, the concentrations of all four generations are halved (See Figure 5B, detail (a)), while the fifth generation has a corresponding high initial value (Figure 5B). Detail (a) in Figure 5B, Figure 6B, Figure 7B, and Figure 8B highlights the beginning of the last generation, to the birth of which all the other generations contribute synchronously, which determines a much more visible increase in concentration than in the case of the previous births. In conclusion, the formation of a new generation causes a reduction by half of the mass of the generations that contribute to the formation of the new generation. After the fifth generation (Figure 5B), the strain reaches the stationary phase, followed by the decay by death of all generations, concomitant with substrate depletion (Figure 5C). Due to the cellular lysis, the dead cells become C and N sources for the living cells (see the discontinuities on the substrate depleting curve, and its final delayed small increase).

When MRS is used as a substrate, the lag period is considerably reduced (5 h, Figure 6A), the final biomass concentration is much higher (Figure 6A), and, in accordance with the experimental results, the stationary phase is longer (Figure 6B,C). Here, also, five generations have time to appear. It must be emphasized that, due to substrate depletion, the cells cannot grow, and, therefore, they are not able to divide. Also, due to the slow but steady substrate formation by cellular lysis, the cells do not die noticeably, like in the previous case.

*L. acidophilus* cultivated on SSCM presents a short lag period of only 5 h (Figure 7A) compared to cultivation on MRS, where the lag period is 20 h (Figure 8A). Because glucose is an easy assimilable source, when *L. acidophilus* is cultivated on SSCM, after the fourth generation, the living cells reach the stationary phase (Figure 7B) and the accommodation to substrate is more rapid (Figure 7C), but the biomass concentration is smaller. When cultivated on MRS, *L. acidophilus* reaches five generations, after which the living cells attain a complete stationary phase (Figure 8A). The substrate is more efficiently consumed (Figure 8C), and the biomass concentration is higher in comparison with cultivation on SSCM. Again, there is a balance between substrate consumption and appearance due to the cell’s lysis, so cell death is negligible (Figure 7B,C and Figure 8B,C).

**Figure 9:**
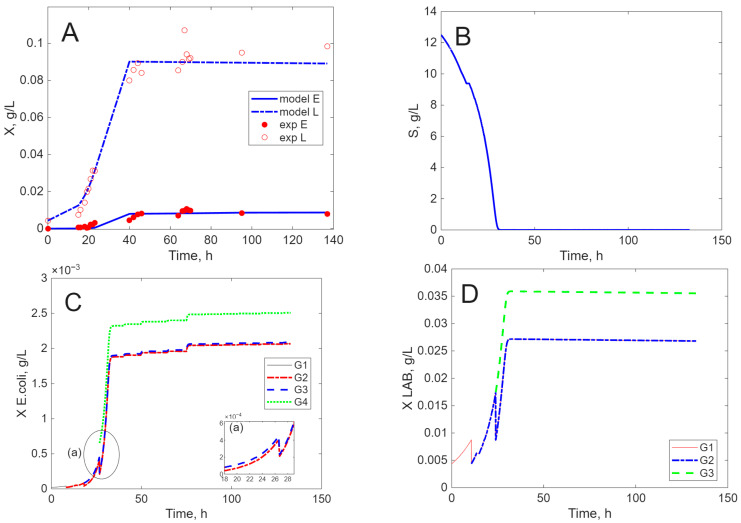
Experimental and simulated results for *L. acidophilus* (1) and *E. coli* (2) on MRS ((**A**)—biomass concentration, (**B**)—substrate concentration, (**C**)—*E. coli* cell generations, and (**D**)—*L. acidophilus* cell generations).

The co-cultivation simulations also show a satisfactory model–experiment agreement, with the results being presented in Figure 9.

The experimental results and model simulation of co-culture cultivation on MRS with simultaneous inoculation show that the microorganisms have different lag periods (longer for *E. coli*). The biomass concentration of *E. coli* is smaller (Figure 9A), and this strain needs four generations to reach the stationary growth phase (Figure 9C). Due to the different life cycles of these cells, *L. acidophilus* needs only three generations (Figure 9D) to enter the stationary phase. The standard substrate concentration (lactose for MRS) is rapidly decreased (Figure 9B), with the growth of the microorganisms being ensured by cellular lysis of the dead cells.

## 3. Discussion

This experimental research aimed to study the population dynamics in a mixed culture of two bacterial strains, *E. coli* and *L. acidophilus*. These two strains were chosen because, in addition to morphological differences that allow for differentiation by Gram staining, *E. coli* is a pathogen with a wide range of distribution, while *L. acidophilus* is a common probiotic. The study analyzed the behavior of the two strains in pure culture on two different culture media (one general—synthetic SSCM—and the other specific for the differentiation of lactic acid bacteria—MRS).

Synchronous cultivation on MRS shows a similar development of the two strains until the exponential phase, when the cycles of domination of one of the strains are pronounced, double compared to the other, within a very short time, considering the duration of the whole process. In the stationary phase, *L. acidophilus* becomes the dominant microorganism.

Cultivation under shifted conditions by inoculating *E. coli* after the lag of the *L. acidophilus* strain finished indicates that the probiotic bacteria maintain the advantage gained during the lag phase (changes in the enzymatic system), controlling the concentration of the pathogenic strain and maintaining it at lower values throughout the entire process.

For in vitro cultivation on the synthetic media, the inhibitory mechanism of *L. acidophilus* against *E. coli* is given by the secretion of a small bacteriocin, acidophilin 801 [42], H_2_O_2_, and lactic acid [46,47] in the exponential phase. The presence of the bactericidal substance, secreted by *Lactobacillus* under stress conditions [48,49], negatively influences the growth of both microbial strains. However, once accumulated in dead cells, it allows for a new cell proliferation cycle during the transformation of dead cells into substrate. The acidophilin 801 and lactic acid accumulated concentrations in the stationary phase determine a considerable reduction in *E. coli* population in all the cultivation cases studied in this work. Metabolites produced by *E. coli* with a possible negative effect on lactobacilli—toxin IV, Shiga-like toxins [50,51], and ethanol—do not reach the concentration levels necessary to curb the proliferation of lactic bacteria.

Of the 3760 metabolites produced by *E. coli* [52], those likely to affect the growth of *Lactobacillus* are ethanol and Shiga-like toxins. Under micro aeration conditions, both micro-organisms produce acids (mostly acetic—both, lactic—*Lactobacillus* mainly) and both have the necessary metabolic pathways to resist a lower pH [53,54]. However, probiotic bacteria exert influence on commensal microorganisms by producing bacteriocins, hydrogen peroxide, and lactic acid. Through the conjugated action of these substances and in conditions of discontinuous cultivation, *Lactobacillus* succeeds in gaining supremacy over the fermentation environment in the stationary phase of culture development [55].

The model predicts that the *E. coli* concentration is kept at lower values because of both cellular death and inhibition determined by the lactic acid produced by *L. acidophilus*. This suggests that future investigations should focus on lactic acid concentration determinations to allow for a better quantification of its effect on the product yield and inhibition of the *L. acidophilus* process.

## 4. Materials and Methods

### 4.1. Materials

The bacterial strains used are *Lactobacillus acidophilus* (ATCC 4796) from the lactobacillus bacterial (LAB) group and *Escherichia coli* (K12-MG1655), and both strains are from the microbiology laboratory collection, preserved on agar slant. Two culture media were used in the study: a culture medium specific to lactic bacteria MRS Broth (Carl Roth, Karlsruhe, Germany) and a general medium with a single source of carbon (SSCM)—glucose and mineral salts. The SSCM medium is obtain from the following three fractions, each with different compositions and specific behavior in sterilization conditions: *fraction 1*: 6 g Na_2_HPO_4_; 3 g KH_2_PO_4_; 0.5 g NaCl; 1 g of NH_4_Cl (g·L^−1^) (Carl Roth, Germany)—sterilized by autoclaving at 121 °C; *fraction 2*: 5 mL glucose 20%; 2 mL MgSO_4_ 1 M; (Carl Roth, Germany)—sterilized by filtration; *fraction 3*: 5 g EDTA; 0.8 g FeCl_3_; 0.05 g ZnCl_2_; 0.0001 g CuCl_2_; 0.0001 g CoCl_2_; 0.0001 g H_3_BO_3_; 0.016 g MnCl_2_. (Sigma-Aldrich, Darmstadt, Germany)—sterilized by filtration, with an adjustable pH to 7 units.

For both microorganisms, the inoculum was grown on their specific medium (MRS or SSCM) to ensure proper and equal accommodation of the bacterial strains to the bioreactor conditions.

### 4.2. Equipment and Process Parameters

The experiments were performed in a Sartorius BIOSTAT^®^ A Plus bioreactor of 2 L with a working volume of 1.5 L (Sartorius, Göttingen, Germany). The auxiliary equipment used to perform online measurements of the optical density (OD) of the cultivation medium was Optical Spectrophotometer Ocean Optics Inc. Jazz (UV-VIS), with an immersion probe (Ocean Optics, Orlando, FL, USA).

The cell viability and Gram differentiation were assessed with an Apogee Flow System A50 Universal (Apogee, Kent, UK) flow cytometer.

The bioreactor was provided with the following: air with a flow rate equal to or less than 0.33 L/min (for occasional micro aeration); mechanical stirring (blade impeller), with the normal speed set to 120 rpm; thermal control (37 °C set point); a pH meter, for pH monitoring of the culture medium; and an oxygen meter (pO_2_ sensor) to measure the dissolved oxygen concentration in the fermentation medium, allowing for the identification of metabolic stages. The variation in operating parameters corresponding to the batch mixed cultivation of both microorganisms is represented in Figure S7 in the Supplementary Material.

### 4.3. Methods of Analysis

*Substrate concentration.* For total reduction sugar determination, a colorimetric technique was used to estimate the substrate concentration of the cultivation media using the 3,5-dinitro salicylic acid (DNS) method. The DNS solution (Carl Roth, Germany) is yellow in color, but after reducing sugars, its color changes to dark red, corresponding to the formation of 3-amino-5-nitro salicylic acid. This method is based on spectrophotometric measurement at 540 nm and the use of a calibration curve (with different glucose concentrations) to estimate the reducing sugar content [56].

*Cell growth determination.* Cell growth induces intensification of culture medium turbidity in the discontinuous bioreactor, which was monitored with the aid of the optical probe coupled to the spectrophotometer, at a wavelength of 600 nm.

*Cell viability and differentiation.* Cell viability was determined by flow cytometry measurements using specific fluorescence markers to differentiate between the viable cells and those who have died because of cell interactions and/or life cycle. The staining technique for both cell viability and Gram differentiation is presented in the Supplementary Information. The fluorescence markers used included Propidium Iodide (PI), Sybr Green I (SG) and Hexidium Iodide (HI), which were purchased from Thermo Fischer Scientific-Invitrogen, Waltham, MA, USA, and the sample preparation and staining technique followed the proper instructions, correlating the time of contact of the dye with the analyzed sample type [57].

### 4.4. Mathematical Modeling

This study aimed to describe the growth of two bacterial species on the same or different substrates together with the interactions between these two bacterial populations. The mathematical model, based upon a segregated approach to capturing bacterial kinetics, was implemented in MATLAB^TM^ 2025b (Mathworks, Natick, MA, USA).

The model is conceptually based on a previous work [43], considering a cell’s birth as a discrete event, assuming that when cells reach a certain mass during growth, they divide in half. After division, the mother cell continues its timeline, while the daughter starts its own timeline. The process of aging (accumulation of damaged genetic material in mother cell) is disregarded in the current version of the mathematical model, but it can be easily added. Therefore, mother and daughter will then grow and reach another division step, when they give birth to two identical daughters (not aging), with these progenies forming a new cluster. Each new division will generate a new cluster of new birth cells, which start their new timeline, while the old clusters continue theirs. This way, a temporal segregation appears, together with the synchronicity of the population [43]. To the knowledge of the authors, such kinds of models have not been published in the literature thus far.

The proposed mathematical model is based upon the mass balance of (i) *E. coli* alive cells; (ii) *L. acidophilus* alive cells; (iii) dead biomass (no matter from which of the two strains it comes); and (iv) substrate. As biomass growth proceeds and division events occur, the number of clusters of living cells with the same age will increase accordingly.

For the growth processes, we assumed that cells follow a simple Monod kinetic, considering a single growth-limiting substrate. For cell decay, we assumed a first-order process with respect to their actual concentration, as presented in Equation (1), as follows:

(1) $$\frac{dX_{i}^{k}}{dt}=\mu_{X_{i},\max}\cdot \frac{S}{K_{S-X_{i}}+S}\cdot X_{i}^{k}-k_{d}^{X_{m}}\cdot X_{i}^{k}$$

where *X_i_* is the cell concentration for strain *i*, g·L^−1^; t—time, s; *µ_Xi,max_*—maximum growth specific rate, h^−1^; *S*—substrate concentration, g·L^−1^; *K_S-Xi_*—the affinity constant, g·L^−1^; and $k_{d}^{X_{m}}$—cell death constant, h^−1^. Furthermore, the index *k* indicates the current generation of cells and the index *i*, the entity to which they belong, i.e., *E. coli* (n) or *L. acidophilus* (m).

Dead cells suffer a lysis process, which is assumed to be the same irrespective of the provenience of the former. During the lysis process, dead cells are completely transformed into substrate by a time delay process, for which the rate of is denoted $v_{lys}$ and can be expressed as presented in Equation (2), as follows:

(2) $$v_{lys}=k_{s}\cdot X_{d}(t-\theta )$$

where *k_s_* is the rate constant of lysis process, h^−1^; *X_d_*—the concentration of dead cells, g·L^−1^ at time *t* − *θ*; and *θ*—the lag time for the dead cells lysis into substrate, h.

Therefore, the rate of accumulation of dead cells can be expressed as follows:

(3) $$\frac{dX_{d}}{dt}=\sum_{j=1}^{M}(k_{d}^{X_{m}}\cdot X_{m}^{j})+\sum_{j=1}^{N}(k_{d}^{X_{n}}\cdot X_{n}^{j})-k_{s}\cdot X_{d}(t-\theta )$$

where the first and second terms represent the sum of dead cells from all clusters for *E. coli (m)* and *L. acidophilus (n)*, respectively. It must be emphasized that different growth rates for the two strains will result in different numbers of clusters.

For substrate consumption, a similar expression, Equation (4), can be written, since all cells consume the substrate, as follows:

(4) $$\frac{dS}{dt}=-\sum_{j=1}^{M}(\frac{\mu_{X_{m,\max}}}{Y_{XS-X_{m}}}\cdot \frac{S}{K_{S-X_{m}}+S}\cdot X_{m}^{j})-\sum_{j=1}^{N}(\frac{\mu_{X_{n,\max}}}{Y_{XS-X_{n}}}\cdot \frac{S}{K_{S-X_{n}}+S}\cdot X_{n}^{j})+k_{s}\cdot X_{d}(t-\theta )$$

For the co-culture experiments, the mathematical model was slightly adjusted to account for the lactic acid production and subsequent inhibition of *E. coli* growth, and expressed as follows in Equation (5):

(5) $$\frac{dX_{n}^{k}}{dt}=\mu_{Xn,\max}\cdot \frac{S}{K_{S-X_{n}}+S+\frac{P}{K_{P}}}\cdot X_{n}^{k}-k_{d}^{X_{n}}\cdot X_{n}^{k}$$

where *P* denotes the lactic acid concentration, g·L^−1^, and *K_P_*—the dimensionless inhibition constant. Thus, lactic acid is a supplemental species, added to the model, for which the accumulation rate (neglecting the maintenance phase) is as follows:

(6) $$\frac{dP}{dt}=\sum_{j=1}^{N}(\frac{\mu_{Xn,\max}}{Y_{XP-X_{n}}}\cdot \frac{S}{K_{S-X_{m}}+S}\cdot X_{n}^{j})$$

where *Y_XP−Xn_* represents the lactic acid yield, expressed as g biomass/g lactic acid.

The mathematical model was solved according to the details given in Figure S1, which is detailed in “S1. Solving the mathematical model algorithm”, Supplementary Information.

## 5. Conclusions

The current findings indicate that the secondary metabolites produced by the two microorganisms, depending on the stage of population development in batch cultivation, influence the relationship of domination between the two bacterial strains. The use of simple culture media and the flow cytometric technique allowed for obtaining experimental data for the development of a mathematical model to characterize bacterial growth in mixed culture.

Mathematical modeling allows for highlighting the general trend of cellular evolution in individual and mixed culture, attenuating the concentration leaps specific to the cyclical evolution of cell populations. It could be refined considering death, as well, as a discrete event, instead of a continuous first-order decay process.

## Figures and Tables

**Table 1:** Kinetic parameters of the mathematical model and the objective functions.

Strain	Media	K_S-X_/ (gL^−1^)	Y_xs_ × 10^3^/ (g g^−1^)	µ_max_/ h^−1^	k_S_/ h^−1^	k_d_ × 10^5^/ h^−1^	F_OB_
*E. coli*	SSCM	79.8 ± 3.2	67 ± 4.1	1.02 ± 0.047	0.211 ± 0.001	480 ± 29	(112 ± 5.6) × 10^−3^
	MRS	70.8 ± 3.4	78 ± 3.8	1.88 ± 0.08	(74 ± 3.7) × 10^−3^	2.32 ± 0.12	(140 ± 6.5) × 10^−3^
*L. acidophilus*	SSCM	1.12 × 10^4^ ± 540	63 ± 3.1	147 ± 7.6	0.827 ± 0.038	(9.72 ± 0.5) × 10^−2^	0.092 ± 0.048
	MRS	883 ± 43.5	92 ± 4.3	4.38 ± 0.21	0.065 ± 0.004	(3.68 ± 0.18) × 10^−2^	0.095 ± 0.051
*E. coli* and *L. acidophilus*	MRS	(20 ± 0.9) × 10^−4^	(6.18 ± 0.34) × 10^3^	(87 ± 4.3) × 10^−3^	(14 ± 0.66) × 10^−2^	1.61 ± 0.78	0.29 ± 0.014
		(706 ± 35) × 10^−3^	3.6 ± 0.18	(63.9 ± 4) × 10^−3^		4.54 ± 0.23	

## Data Availability

The original contributions presented in this study are included in the article/Supplementary Material. Further inquiries can be directed to the corresponding author.

## Supplementary Materials

The following supporting information can be downloaded at: https://www.mdpi.com/article/10.3390/ijms262311493/s1.

## Nomenclature

*K*	(g·L^−1^) affinity constant
k	(h^−1^) velocity constant
S	(g·L^−1^) substrate concentration
*t*	(h) time
X	(g·L^−1^) cell concentration
P	(g·L^−1^) lactic acid concentration
Y_XS_	(g_cel_·g^−1^_substrate_) transformation yield
**Greek Letters**	
*μ*	(s^−1^) specific growth velocity
*v*	(s^−1^) velocity of dead cell transformation
*θ*	(s) time delay for the dead cell transformation
**Subscripts**	
d	dead
k, l	current generation
n	*E. coli* population
m	*L. acidophilus* population
