# Supplementary material for: Mathematical Modeling of Escherichia coli and Lactobacillus acidophilus Growth Based on Experimental Mixed Batch Cultivation
Source: Int J Mol Sci. 2025 Nov 27;26(23):11493. doi: 10.3390/ijms262311493 (PMC12692680; doi:10.3390/ijms262311493)
Supplement: Supplementary file 1 [file ijms-26-11493-s001.zip › ijms-3944488-supplementary.pdf]

Supplementary Information

Mathematical modelling of *Escherichia coli* and *Lactobacillus acidophilus* growth based on experimental mixed batch cultivation

Gabriela Isopencu[1], Valentina Gogulancea[2], Vasile Lavric[1], Ionut Banu[1]

[1] *Department of Chemical and Biochemical Engineering, National University of Science and Technology POLITEHNICA Bucharest, 1-7 Gh. Polizu, 011061 Bucharest, Romania; gabriela.isopencu@upb.ro (G.I.), vasile.lavric@upb.ro (V.L.), ionut.banu@upb.ro (I.B.)*

[2] *School of Computing, Eng & Intel. Sys, Ulster University, Derry~Londonderry campus, UK, v.gogulancea@ulster.ac.uk (V.G.)*

*Corresponding author, e-mail: ionut.banu@upb.ro

S1. Solving the mathematical model algorithm

The model is used for regression analysis over the experimental data, the optimization of the objective function being done via the genetic algorithm package implemented in Matlab™ 2025b (Mathworks, Natick, MA, USA) to determine the kinetic parameters of the two species, grown individually on different cultivation media. The structure of the solving algorithm is described in **Figure S1**.

The data are fed to the main program, which calls the Genetic Algorithms, where the objective function (the mean of the squared residuals model-experiment) that evaluates the model-experiment departure is minimized. The experimental interval is split into smaller intervals to account for the times of experimental readings. For each subinterval, the system of ordinary differential equations is solved, using Matlab™ (Mathworks, Natick, MA, USA) integration routine *ode15s.* After each successful integration step, a cell's division check is performed in an auxiliary function and if conditions for division are fulfilled, the concentrations are adjusted, and a new cluster of cells is formed, for the newborn offsprings.

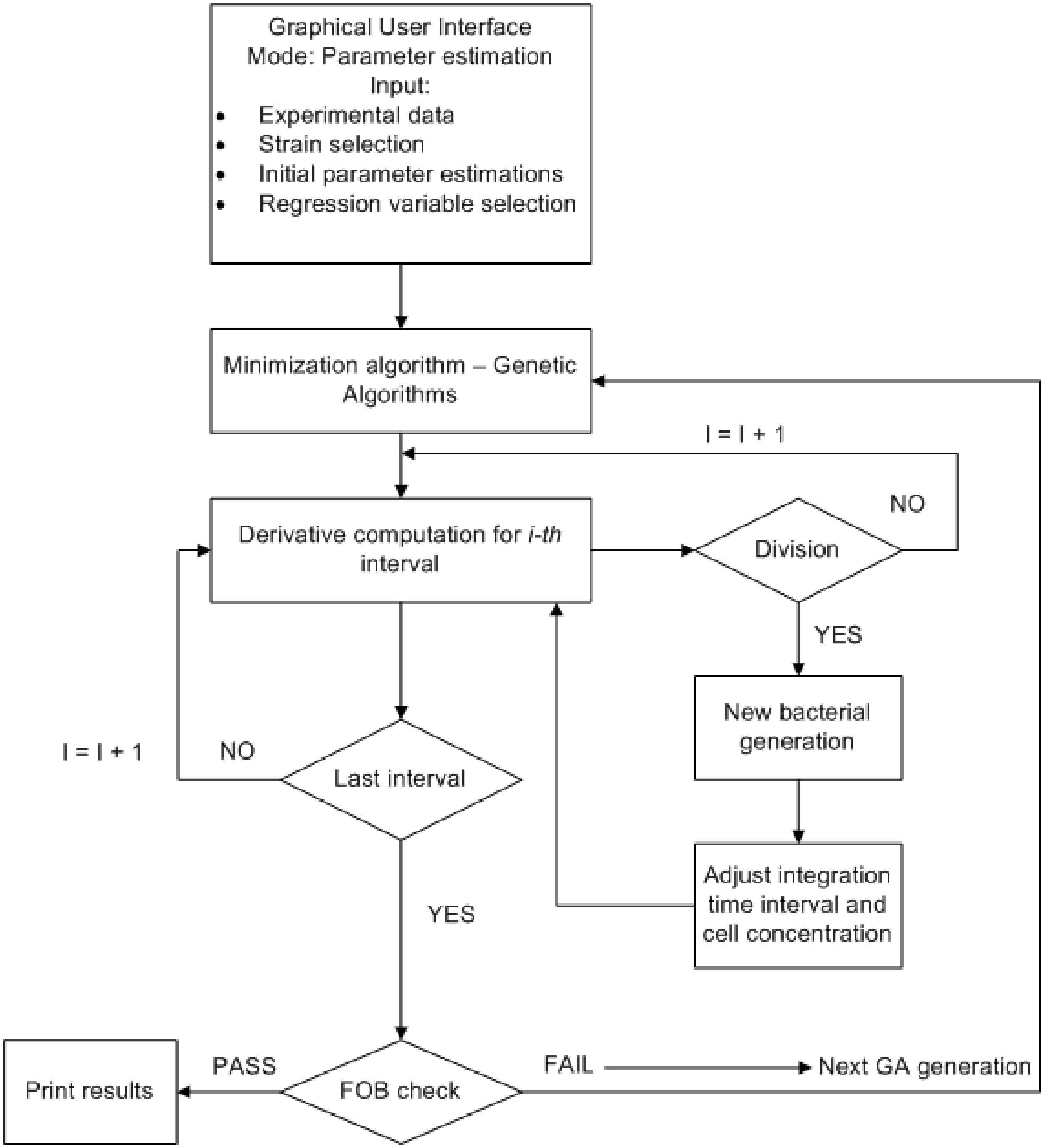

Figure S1. The structure of the solving algorithm implemented in Matlab™ (Mathworks, Natick, MA, USA)

S2. Cell viability – protocol and results

Cell viability was determined by flow cytometry measurements using specific fluorescence markers to differentiate between the viable cells and those who have suffered injury as a result of cell interactions and/or life cycle.

Reagents used as fluorescent markers are propidium iodide (PI) used to identify dead cells, Syber Green (SRG), for live cells and iodide hexide (HI) for Gram differentiation.

The cell sample was diluted 1: 1000 bacteria / mL in sterile distilled water. For each experiment, different types of samples were prepared, depending on the desired analysis, namely:

a. for viability

- white sample: uncolored, allows to find the total number of cells in the sample,
- red sample: colored with PI, used to identify dead cells;
- green sample: stained with SRG, used to identify live cells;
- discrimination sample: stained with equal amounts of SRG and PI to discriminate live cells from dead cells and to identify injured cells.

b. for gram differentiation

- white sample: uncolored, allows to find the total number of cells in the sample,
- red sample: stained with HI, used to identify Gram-positive cells;
- green sample: colored with SRG, used to identify the total number of living cells;
- discrimination sample: stained with equal amounts of SRG and HI for discriminating Gram-negative cells than negative.

Dyeing method: 250 μL of cell sample is pipetted into an Eppendorf tube together with a 2.5 mL fluorescent marker. The tube is shaken vigorously and left in the dark for at least 5 minutes.

The flow cytometer is calibrated for bacterial measurements.

In **Figure S2**, from *Supplementary Information*, are presented some representative images recorded with the flow cytometer for *E. coli* strain on MRS, because this medium is unfavourable for this strain growth. The cytogram with axes LALS vs. SALS represents the total number of microorganisms in sample while the cytogram with axes Org vs. Grn are used for live-dead cell discrimination by marking with those two fluorescent markers mentioned above. Based on the valued obtained for samples collected from culture every 2 hours, the graphs represented in **Figure S3** (a) and (b) in *Supplementary Information* were obtained.

Backing-up the experimental observations on the cell density (turbidity) related to the long duration of lag time when *E. coli* strain was cultivated on MRS, the flow cytometry shows that, during this lag phase, massive cell death occurs, as a result of forced cell adaptation (Figure S3 (b) - *Supplementary Information*), phase followed by normal cell growth.

On the SSCM cultivation media, cell viability is positive, the rate of decay being exceeded by the rate of cellular divisions (shown in Figure S3 (a) - *Supplementary Information)*.

In Figure S4, the cytograms for *L. acidophilus* cultivated on MRS are presented. The complexity of those cytograms appears from the fact that alive lactic bacteria show a natural florescence, but at lower signal strength than the colored sample.

L. acidophilus shows a synchronous variation in cell number during cell growth. On the SCCM media (Figure S5 (a)-*Supplementary Information*), there is a higher concentration of cells alive compared to the dead ones, with some synchronicity in the stationary phase of cellular development. MRS is a specific environment for lactic bacteria growth and isolation, as evidenced by the cell viability curves in Figure S5 (b) - *Supplementary Information*, which indicates a viability threshold at the onset of the stationary growth phase.

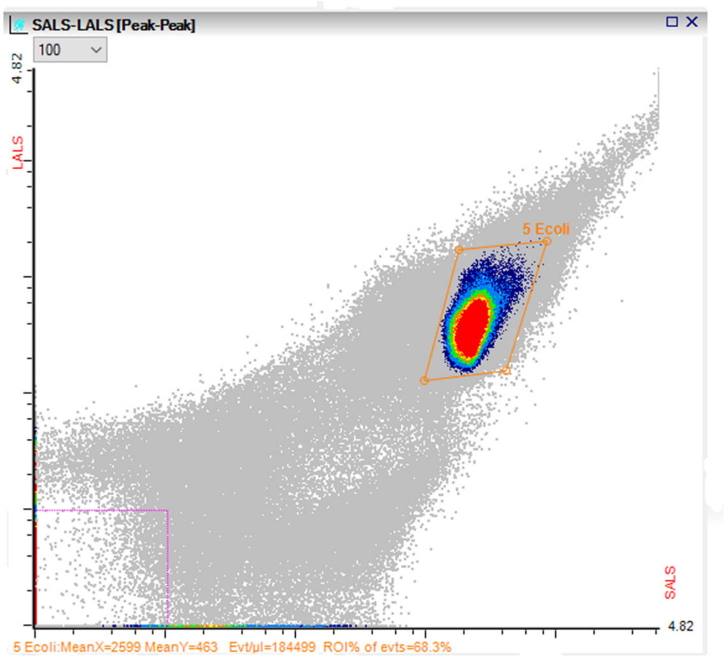

a)

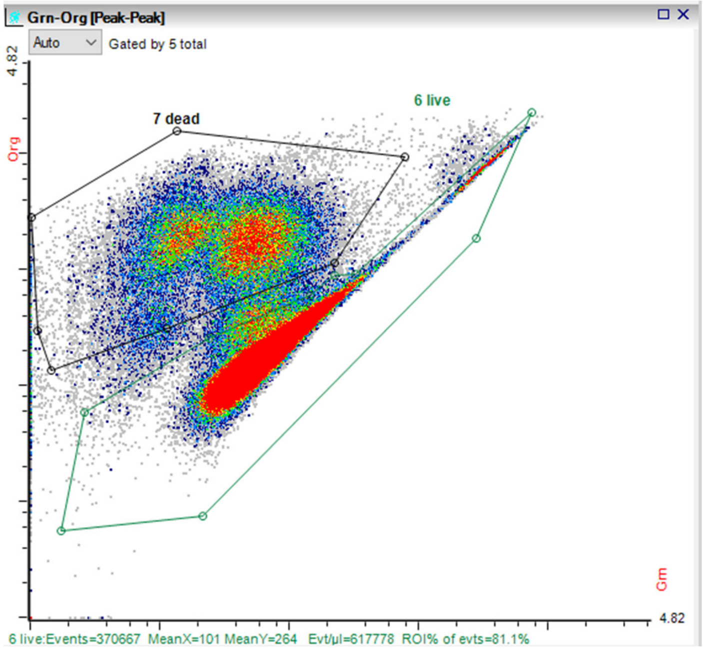

b)

Figure S2. The cytograms for *E coli* strain cultivated on MRS. white probe (a) total cells, and (b) - discrimination probe - for cells viability live/dead cells

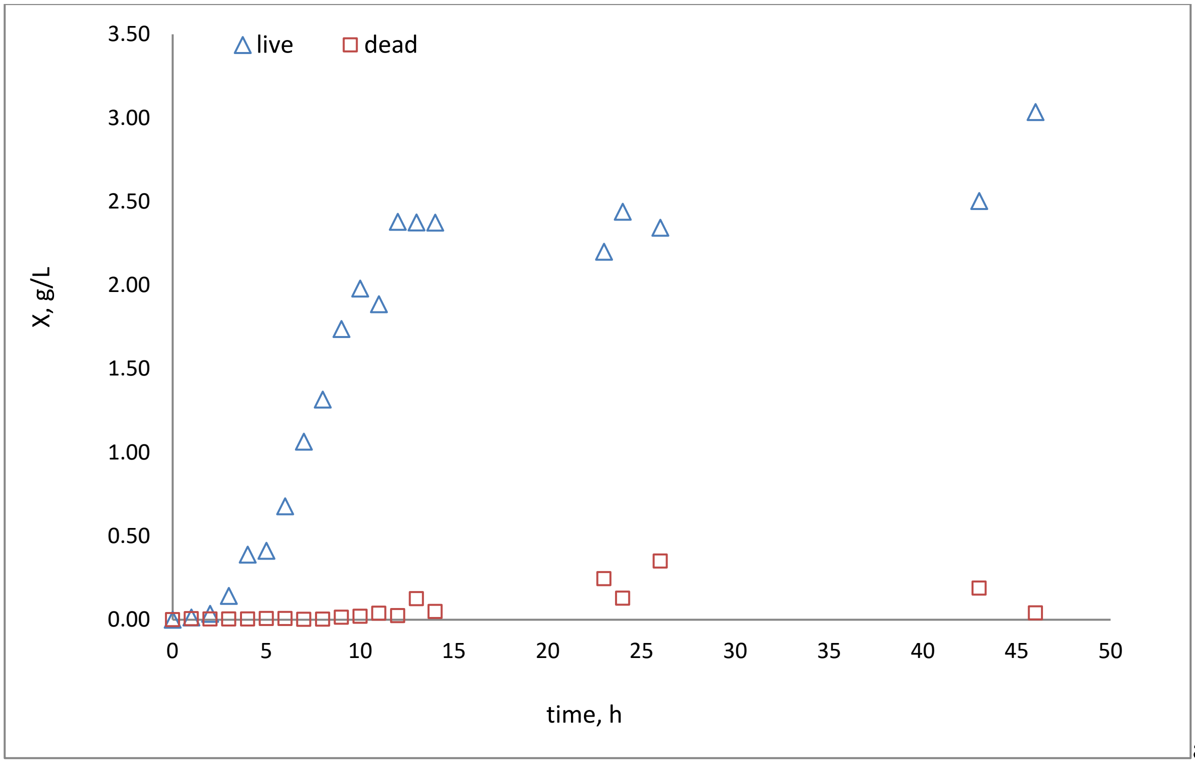

a)

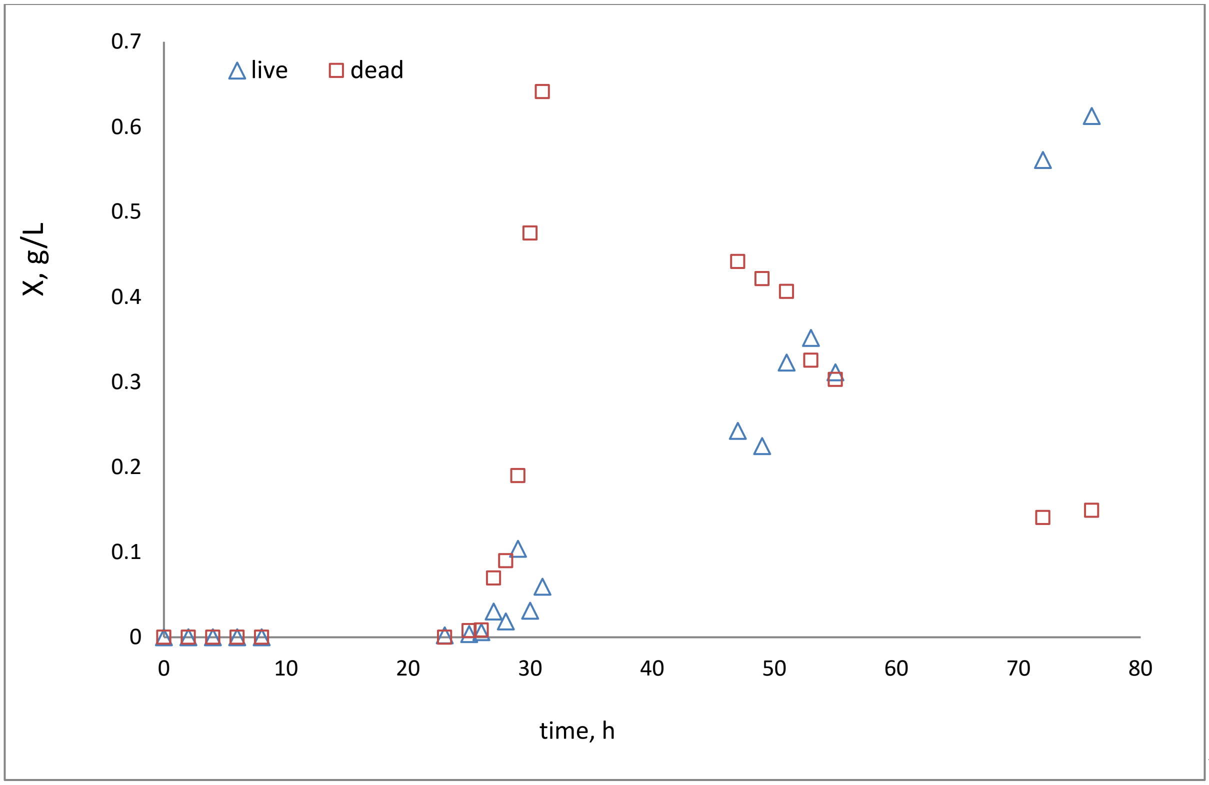

b)

Figure S3. Time variation of the live (blue triangles)/dead (red squares) cells concentration of *E. coli i*n culture on the SSCM (a) and MRS (b)

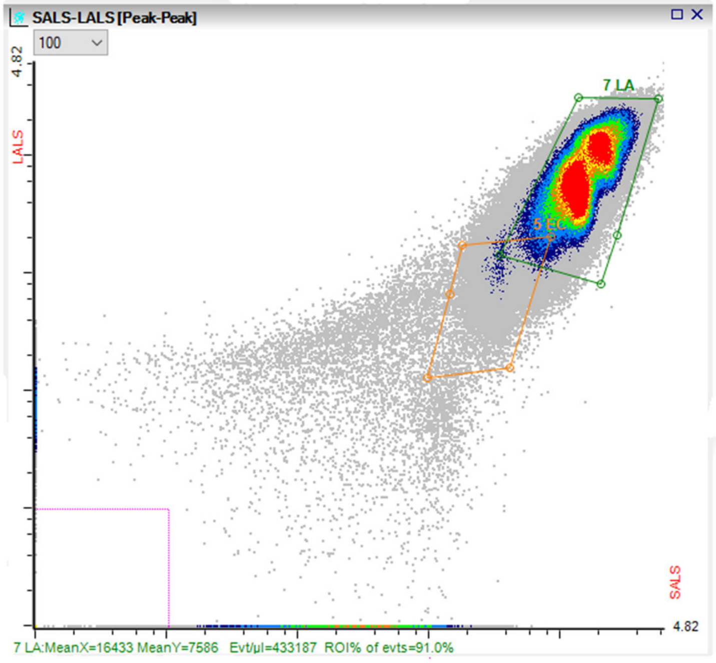

a)

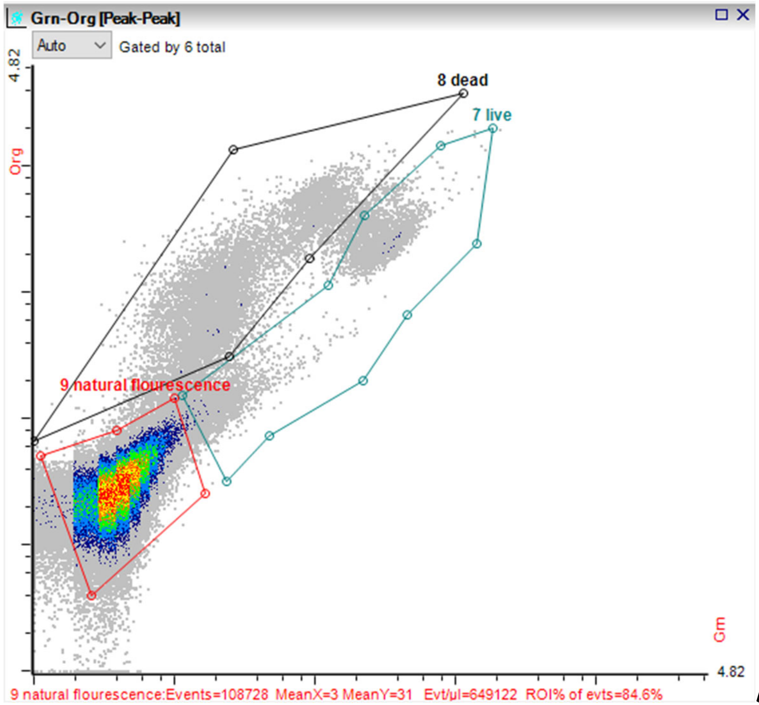

b)

Figure S4. The cytograms for *L. acidophilus* strain cultivated on MRS, white probe (a) - total cells, and (b) - discrimination probe – for cells viability live/dead cells

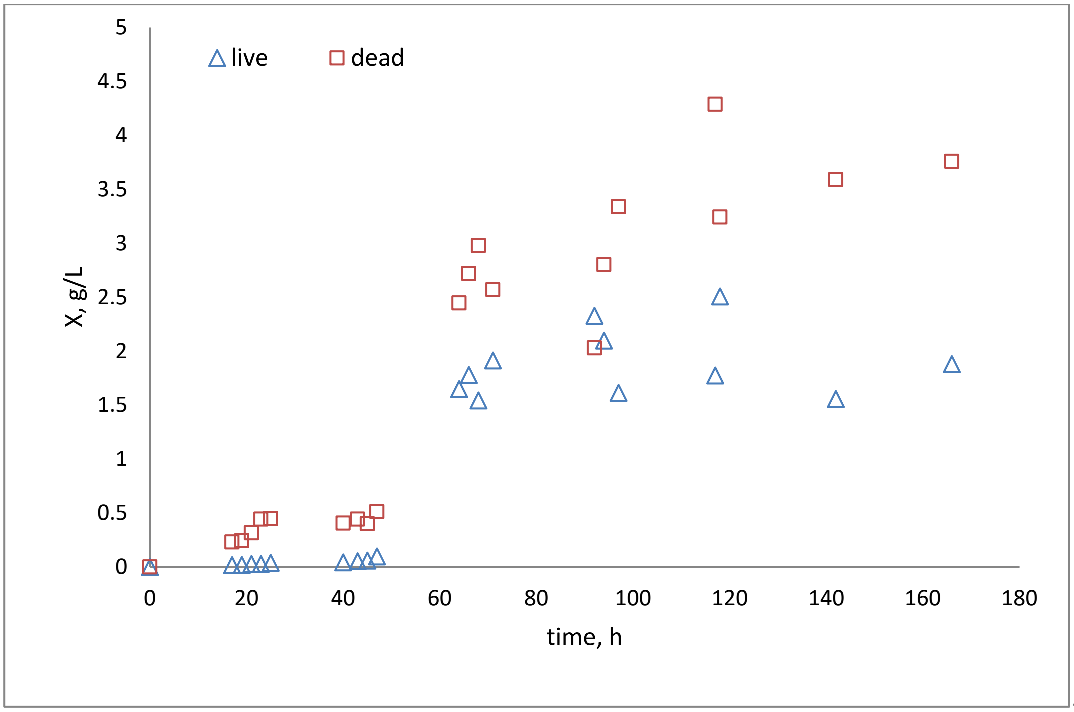

a)

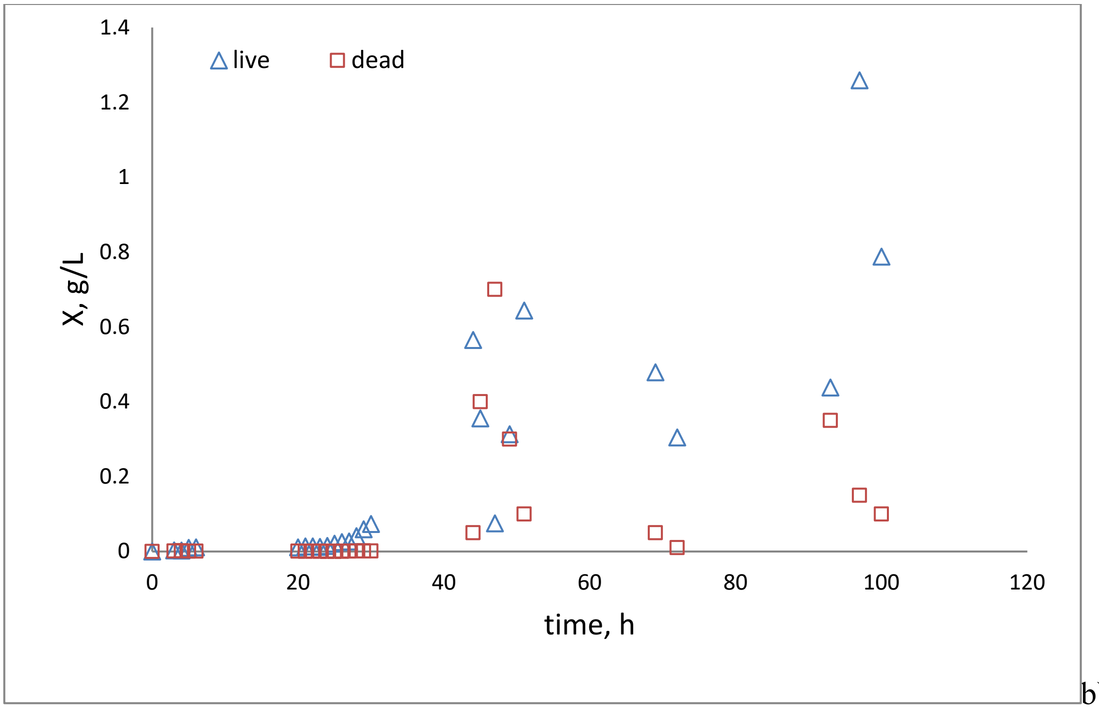

b)

Figure S5. Time variation of the live (blue triangles)/dead (red squares) cells concentration of *L. acidophilus* in culture on the SSCM (a) and MRS (b)

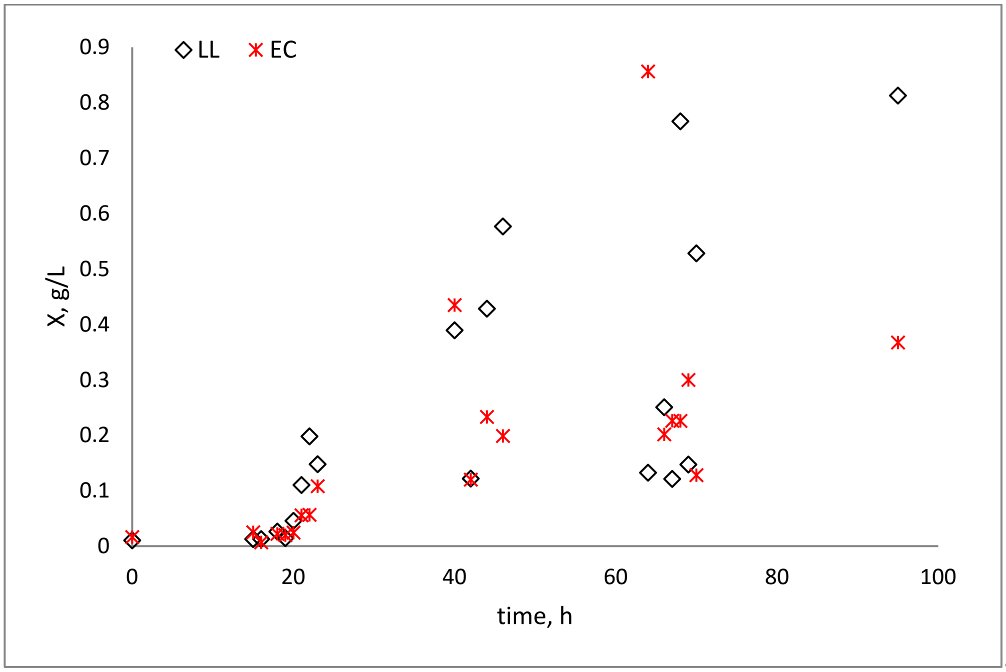

a)

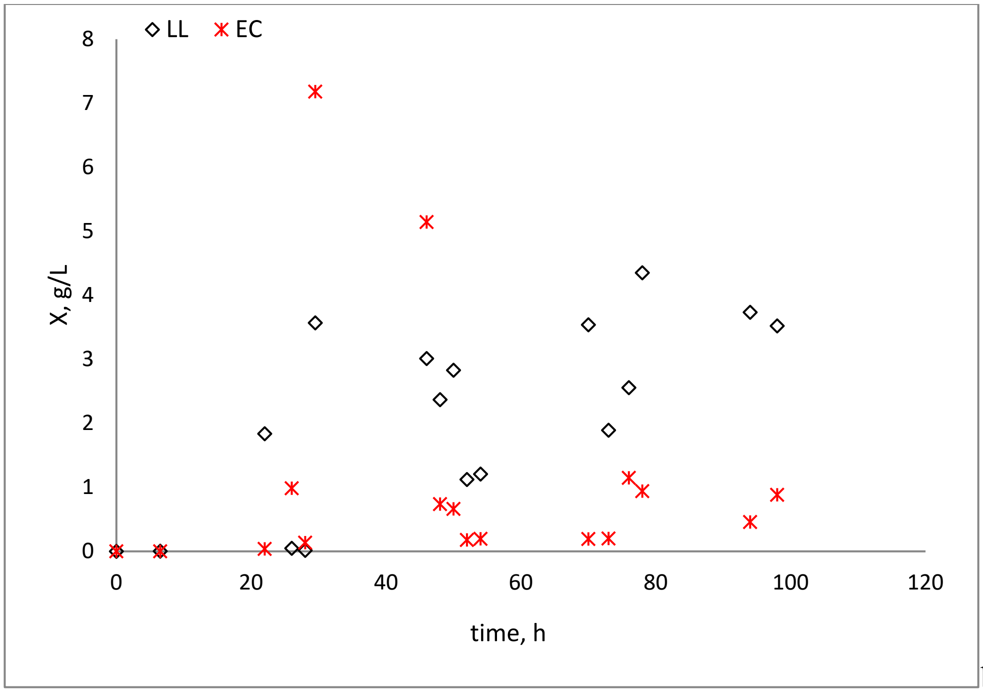

b)

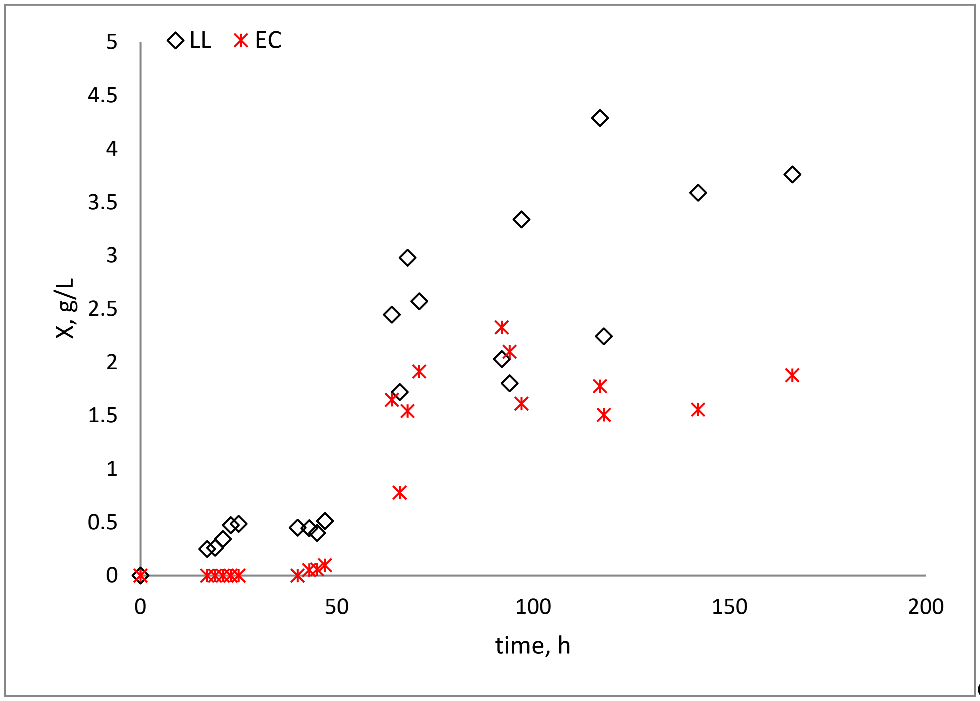

c)

Figure S6. Cell concentration for Gram differentiation in time for mixed culture and (a) *Sync-co* on SSCM, (b) *Sync-co* on MRS, (c) *Lag- sync* on SSCM with highlighting the Gram positive (LL) and Gram negative (EC) bacteria

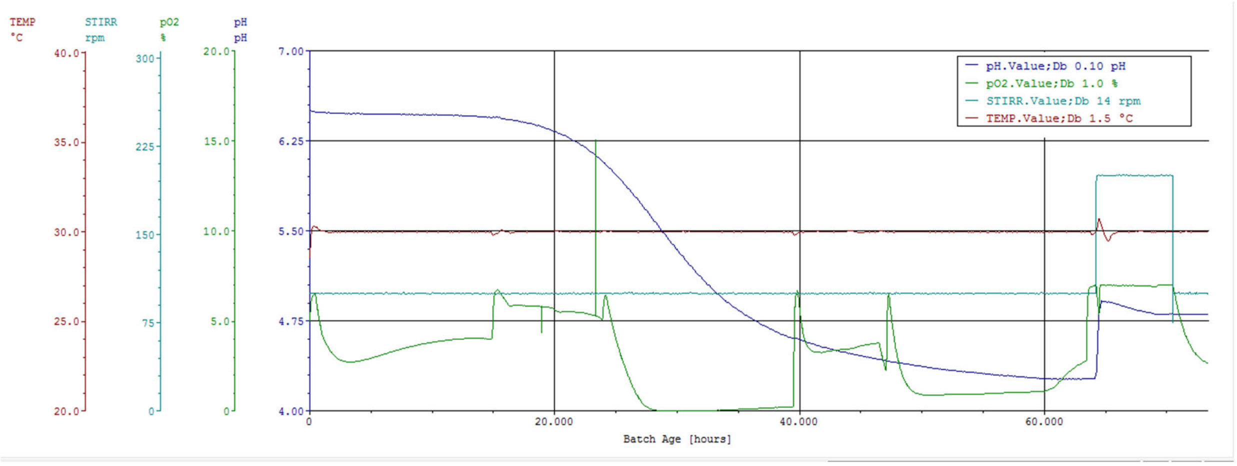

Figure S7. The operating parameters for a typical batch in the bioreactor
